# Efficient *In Vivo* Selection of a Novel Tumor-Associated Peptide from a Phage Display Library

**DOI:** 10.3390/molecules16010900

**Published:** 2011-01-21

**Authors:** Anka N. Veleva, Desh B. Nepal, C. Brandon Frederick, Jacob Schwab, Pamela Lockyer, Hong Yuan, David S. Lalush, Cam Patterson

**Affiliations:** 1 Department of Biomedical Engineering, North Carolina State University, Raleigh, NC 27695, USA; E-Mails: cbfreder@email.unc.edu (C.B.F.); dslalush@unity.ncsu.edu (D.S.L.); 2 McAllister Heart Institute, University of North Carolina, Chapel Hill, NC 27599, USA; E-Mails: dbnepal@gmail.com (D.B.N.); schwab.jacob@gmail.com (J.S.); plockyer@med.unc.edu(P.L.); cpatters@med.unc.edu (C.P.); 3 Biomedical Research Imaging Center, University of North Carolina, Chapel Hill, NC 27599, USA; E-Mail: yuanh@med.unc.edu (H.Y.); 4 Department of Medicine, University of North Carolina, Chapel Hill, NC 27599, USA; 5 Department of Cell and Developmental Biology, University of North Carolina, Chapel Hill, NC 27599, USA; 6 Department of Pharmacology, University of North Carolina, Chapel Hill, NC 27599, USA

**Keywords:** *in vivo* phage display, circulating bone marrow derived tumor homing cells, tumor-associated peptides, targeting neovascular growth, positron emission tomography (PET) imaging

## Abstract

We developed a screening procedure to identify ligands from a phage display random peptide library that are selective for circulating bone marrow derived cells homing to angiogenic tumors. Panning the library on blood outgrowth endothelial cell suspension *in vitro* followed by *in vivo* selection based on homing of bone marrow-bound phage to angiogenic tumors, yielded the peptide QFPPKLTNNSML. Upon intravenous injection phage displaying this peptide homed to Lewis lung carcinoma (LLC) tumors *in vivo* whereas control phage did not localize to tumor tissue. Phage carrying the QFPPKLTNNSML peptide labeled with ^64^Cu radionuclide when administered intravenously into a tumor bearing mouse was detected noninvasively with positron emission tomography (PET) around the tumor. These proof-of-principle experiments demonstrate the ability of the QFPPKLTNNSML peptide to deliver payload (radiolabeled phage conjugates) *in vivo* to sites of ongoing angiogenesis and point to its potential clinical utility in a variety of physiologic and pathologic processes where neovascular growth is a critical component.

## 1. Introduction

The discovery and development of new cancer therapeutic and diagnostic compounds have traditionally relied on (i) isolation from natural sources, e.g., bioactive peptides, (ii) screening of chemical libraries, (iii) computer-based rational design, and (iv) antibody engineering [1]. Phage display peptide libraries provide an alternative source to acquire unique peptide ligands for various cancer-related disease targets. Phage display technology holds great promise to advance cancer diagnosis and treatment. Although numerous peptides have been discovered through screening combinatorial phage libraries only a small subset consist of cancer targeting peptides [2,3]. This is most likely associated with the convoluted processes involved in tumor development and the lack of well-defined tumor markers. Therefore new approaches for selection of tumor targeting peptides from phage libraries informed by knowledge of tumor biology are needed. 

Angiogenesis is considered to play a major role for the growth and dissemination of malignant tumors [4]. Tumors form new blood vessels either from pre-existing mature ones or from *de novo* by recruiting circulating endothelial and hematopoietic precursor cells [5]. Stromal cells of bone marrow (BM) origin have been identified in the vasculature of several pre-clinical models [6,7,8]. Importantly, circulating endothelial precursors have been shown to control the angiogenic switch in mouse lung metastasis [9]. In humans, BM-derived endothelial cells have been detected in patients with multiple myeloma [10], primary breast cancer [11], non-small cell lung cancer [12], and malignant gliomas [13]. Hematopoietic cells have also been shown to home to tumor tissue. Recent evidence indicates that chemokines such as vascular endothelial growth factor (VEGF) promote co-mobilization of circulating endothelial and hematopoietic precursor cells to the tumor vascular bed where they contribute to neovessel formation [14].

As bone marrow derived cells home to tumors, we hypothesized that peptides binding to these cells may be of interest as tools for the specific delivery of diagnostic reagents and anticancer therapeutic compounds. Thus in this study we set to identify peptide ligands from a phage display library that specifically recognize cell populations of BM origin recruited to tumors and to characterize their tumor homing ability *in vivo*. Panning the library using a novel combined *in vitro* and *in vivo* selection protocol yielded the peptide QFPPKLTNNSML. We demonstrated the ability of the QFPPKLTNNSML peptide to deliver payload (radiolabeled phage conjugates) to sites of ongoing angiogenesis in a subcutaneously implanted Lewis lung carcinoma model. To the best of our knowledge this is the first study reporting the use of phage display technology to target tumors through circulating cells.

## 2. Results and Discussion

### 2.1. Peptide library screening

**Figure 1:**
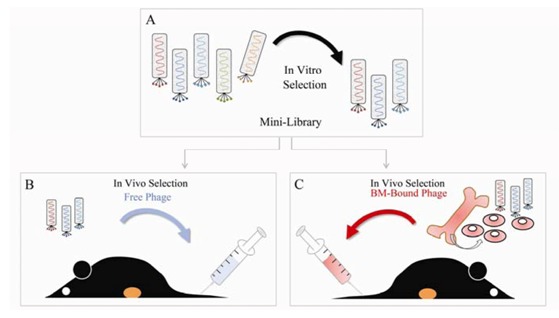
Selection of tumor-associated phage via a combination of *in vitro* and *in vivo* biopanning protocols. (A) A random 12-mer peptide library was pre-selected *in vitro* on HUVEC (one round of negative selection) and on HBOEC (three rounds of positive selection) which resulted in an enriched phage pool, designated as mini-library. The mini-library was used for *in vivo* panning of tumor-associated phage employing two distinct selection schemes. (B) The mini-library was injected i.v. in a LLC tumor bearing mouse. Phage were allowed to circulate for 2 hours and the mouse was perfused with PBS. Then the tumor was excised, the tumor-bound phage pool was amplified in *E. Coli,* and used for another round of biopanning. Total of three *in vivo* selection cycles were conducted utilizing free phage. (C) Freshly isolated bone marrow (BM) cells were labeled with the mini-library. BM-bound phage were injected i.v. into the tail vein of a LLC tumor bearing mouse. After 2 hours of circulation the mouse was perfused with PBS. The phage were rescued from the tumor, amplified, and used to label BM cells for subsequent enrichment cycles. Three rounds of *in vivo* functional selection were performed using BM-bound phage.

During development, hematopoietic and vascular endothelial progenitors originate from a common precursor cell, the hemangioblast, and share several phenotypic characteristics [15]. Here we exploit this shared phenotype and use human blood outgrowth endothelial cells (HBOEC) cultured from peripheral blood mononuclear cells to enrich the phage library before panning on BM cells. This will allow for the discovery of peptides that specifically recognize molecular markers expressed both by endothelial precursors and distinct BM sub-population(s). To distinguish the cell population(s) of BM origin capable of homing to tumors, we introduced a functional feature in our screen: only phage carried by BM cells to angiogenic tumors were propagated and used for subsequent enrichment. Figure 1 depicts a schematic of our selection procedures. 

**Figure 2:**
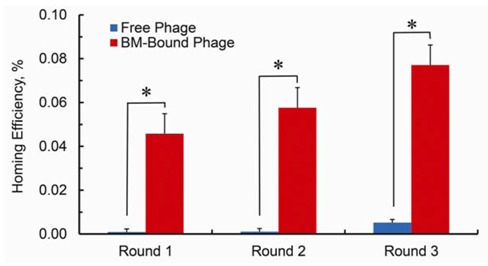
Homing efficiency of the phage pool from each selection cycle, calculated as the ratio of output phage titer to input phage titer multiplied by 100. Using BM cells to deliver phage to tumors improves the efficiency of the selection procedure in the range of 50-fold. The bars show standard error of the mean (s.e.m.) from plating quadruplicates. P values were calculated by Student’s t-test and were considered statistically significant at P < 0.05.

After one round of negative selection on human umbilical vein endothelial cells (HUVEC) to deplete the library of clones that bind to common cell surface receptors and three rounds of positive selection on HBOEC *in vitro*, we generated a mini-library of enriched phage pool for further downstream biopanning (Figure 1A). We utilized two distinct *in vivo* selection protocols in parallel and compared the results: (i) The mini-library was either directly injected intravenously into a mouse bearing subcutaneously implanted LLC tumor (Figure 1B) or (ii) A functional step was employed that consisted of administering BM-bound phage into a mouse bearing subcutaneously implanted LLC tumor (Figure 1C). To enable the latter protocol we isolated murine BM cells from femoral and tibial bones and labeled the cell suspension with the mini-library. Free phage or BM-bound phage were allowed to circulate for 2 hours. Then the mice were perfused through the heart with PBS and the tumors were harvested. Tumor-associated phage pool from each protocol was propagated separately in *E. Coli*, and used in subsequent enrichment cycles. The efficiency of each cycle was quantified as the ratio of the output phage titer to input phage titer multiplied by 100. As seen from Figure 2 using BM cells to deliver phage to tumors improves the efficiency of the selection procedure dramatically. 

**Figure 3:**
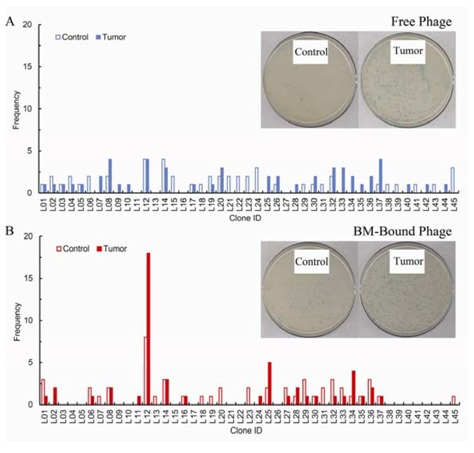
Frequency of sequenced peptide ligands selected either by panning with free phage (A) or by panning using bone marrow-bound phage (B). After three rounds of *in vivo* selection, fifty phage clones homing to tumors and to Matrigel controls from each protocol were randomly chosen for sequencing. Frequency refers to the number of times each phage was isolated out of the total number of phage sequenced. *In vivo* selection with free phage produces no apparent enrichment of particular clones. By stark contrast, three rounds of *in vivo* functional selection, using BM cells to direct the phage pool to the tumor site, are sufficient to identify a dominant amino acid sequence. The inserts in both panels represent typical plates from which phage clones were selected and processed for sequencing.

To rule out library phage binding to the Matrigel matrix which is a component of the implanted tumors, we used a Matrigel plug without LLC cells as a control. Matrigel was injected symmetrically to the LLC tumor in the same animal used for the last round of biopanning. After tumors and the control plugs were excised and bound phage recovered, fifty randomly chosen phage clones resulting from each protocol both for the tumors and the control plugs (200 phage clones in total) were plaque purified and sequenced. Figure 3 presents the phage inserts sequencing distribution profiles for both selection procedures for the tumors and the control plugs. The biopanning protocol with free phage does not produce a noticeable enrichment after three rounds of *in vivo* selection. In contrast to what is seen with free phage, when BM cells are utilized in the panning procedure a dominant phage clone, L12, emerges. The sequencing results in Figure 3 are in agreement with the homing efficiency findings in Figures 2, supporting the observation that the selection procedure based on specific functional attributes, *i.e.,* the target is unique to cancer as these BM-derived circulating cells have the natural ability to localize to tumors, has notable efficiency and produces a phage clone that displays a preferentially enriched amino acid sequence. Regarding specificity, the dominant clone, L12, is recovered from the Matrigel plug with equal frequency to the tumor in the free phage screen, and with highest frequency (8 out of 50 sequenced clones) for the plug in the BM-bound phage screen. However, as shown in Figure 3, we observed that the overall amount of phage homing to tumors in both screens is much higher compared to the amount of phage localizing to the Matrigel controls, *i.e.,* in order to obtain 50 phage clones for sequencing we processed larger amounts of control plugs compared to tumors. Thus we conclude that the selection protocols utilized here are remarkably specific as minimal background phage accumulation to the control plugs has been detected.

### 2.2. Peptide sequence analysis

The peptide sequences that appeared with the highest frequency in the sequenced phage pool are listed in Table 1. Two of the peptides, L12 and L34, were encountered in a previous study that utilized *in vitro* selection protocol for identification of HBOEC-specific peptides [16]. Although these findings confirm our initial hypothesis of shared phenotypic expression between outgrowth endothelial precursors and circulating bone marrow derived cells, neither L12 nor L34 peptide was enriched in the HBOEC-specific phage pool (1 occurrence for each peptide in a pool of 40 sequenced peptides). Furthermore, we searched MimoDB, the newly developed repository of peptides derived by screening of phage display libraries [17], and found that none of the peptides listed in Table 1 has been identified to bind to other targets. We selected the phage clone with the highest frequency of occurrence for further characterization. This phage expresses the peptide sequence QFPPKLTNNSML, denoted also as QFP-peptide. 

**Table 1:** Peptides identified with the highest frequency from the sequenced phage pool.

Code	Peptide sequence (three letter code)	Frequency of occurrence
L12	GLN-PHE-PRO-PRO-LYS-LEU-THR-ASN-ASN-SER-MET-LUE	18
L25	SER-TRY-ASP-ILE-LEU-LYS-PRO-ASN-PRO-GLN-ARG-LEU	5
L34	SER-HIS-GLY-LYS-PRO-PRO-SER-PHE-SER-PRO-TRY-THR	4

Prompted by observations from other studies that peptides isolated by phage display are often functionally active and bind to sites of protein-protein interactions [18,19], we searched the non-redundant protein database (**nr**) at NCBI using the BLASTp tool to find potential homologies to the QFP-peptide [20]. BLASTp alignments with algorithm parameters adjusted to identify short and nearly exact matches, revealed a number of similarities to proteins with cell membrane or extracellular function (see Table 2). Human immunodeficiency virus type 1 enhancer binding protein also recognized as major histocompatibility complex binding protein 1 is known to mediate immune evasion and promote viral persistence by down regulating major histocompatibility complex in peripheral blood mononuclear cells [21]. Serine protease 55 (PRSS55) is a member of a group of membrane anchored chemotrypsin (S1)-like serine proteases that have a role in normal homeostasis as well as in pathology of diseases such as cancer. Homology with the QFP-peptide occurs at the hydrophobic part of the C-terminus of PRSS55 which provides basis for cell membrane anchoring [22]. Cubilin (3623 aa protein) acts as a receptor for intrinsic factor-vitamin B12 (cobaltamin) complexes. Limited tissue expression of cubilin and its putative importance for cell cancer growth have motivated studies to explore cubilin as a new target for the delivery of organometallic B12-based conjugates for cancer diagnosis and treatment [23]. Finally, we have found through analysis for conserved domains that the KLTNNS region of the integrin β2 aligns with the QFP-peptide [24]. Βeta-2 integrins are known to play an important role in the leukocyte-endothelial interactions [25]. Recent studies indicate that circulating tumor cells bind in increased numbers to cytokine activated endothelium in a process reminiscent with of leukocyte-endothelial adhesion [26]. Of note, the extracellular matrix components of Matrigel in the LLC tumors may provide ligands for β2 integrin mediated interactions thus potentially causing off-specificity binding. Whether the QFP-peptide actually participates in the functional interactions identified through bioinformatics analysis and the specific molecular mechanisms involved remains to be further investigated.

**Table 2:** Identified sequence homologies to the QFPPKLTNNSML peptide obtained by searching the non-redundant (**nr)** NCBI protein database performing local sequence alignment. The underlined amino acids correspond to exact alignment of the protein sequences with the new peptide. The numbering on both ends of the sequences indicates the region where homology occurs.

Protein Sequence	Protein Name	Accession
233—PPKLKNSSM –241	Human Immunodeficiency Virus Type 1 Enhancer Binding Protein	AAI 40817.1
227—PKLTKN-ML –234	Serine Protease 55 (PRSS55)	NP_001183949
3118—PPNVKSSNNSML – 3129	Cubilin (intrinsic factor cobalamin receptor)	EAN 86221.1
187—KLTNNS –192	Integrin 2	EAX 09383

### 2.3. Characterization of QFP-phage clone binding to LLC tumors by plaque assay

**Figure 4:**
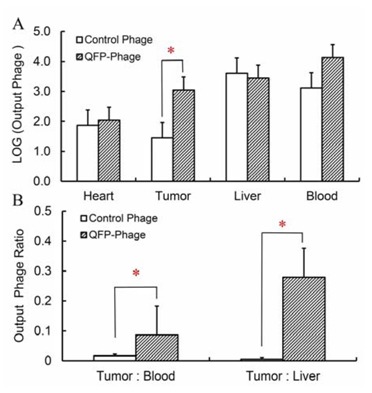
(A) Localization of QFP- and control phage to LLC tumors, heart, liver and blood at 2 hours post-injection of BM cells labeled with phage. Statistically higher amount of QFP-phage was detected in LLC compared to a control insertless phage. (B) Tumor-to-blood and tumor-to-liver ratio for QFP- and control phage. Both ratios are statistically higher for QFP-phage compared to the control (P < 0.05).

QFP-phage was grown to a high titer and used for labeling of freshly isolated BM cells. Phage-BM cell complexes were re-suspended in PBS and injected intravenously into LLC tumor bearing mice (n = 3). Experiments with control non-targeted phage were performed in parallel. After 2 hours of circulation blood was collected, the mouse perfused with PBS, and heart, tumor and liver harvested. The amount of phage in blood and each tissue was determined by plaque assay by counting the number of infectious phage particles. As shown in Figure 4, liver and blood retained the most phage and heart retained the least at 2 hours post-injection. The amount of QFP-phage in the tumor was statistically higher than the amount of non-targeted phage. Furthermore, tumor-to-liver and tumor-to-blood ratio were significantly higher for QFP-phage compared to the control.

### 2.4. Noninvasive quantitation of in vivo peptide binding to LLC tumors by positron emission tomography

**Figure 5:**
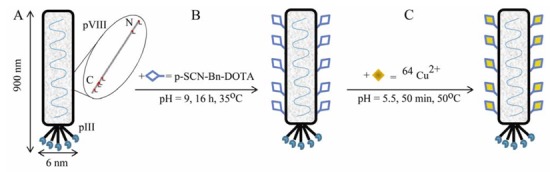
Design and synthesis of M13 phage-based molecularly targeted imaging platform for noninvasive assessment and visualization of *in vivo* peptide binding by positron emission tomography (PET). (A) M13 bacteriophage is a long filamentous particle approx. 6 nm in diameter and 900 nm in length. The viral genome is encapsulated in approx. 2700 copies of the major coat protein pVIII. The minor coat protein pIII that caps the particle is engineered to display five copies of a tumor-avid QFPPKLTNNSML peptide. Five lysine groups (Lys 8, 40, 43, 44, 48) and one N-terminal amino group (Ala 1) on each pVIII subunit are available for chemical modification [28]. (B) Bifunctional p-SCN-Bn-DOTA derivative is added to a 1×10^11^ pfu phage in conjugation buffer at pH = 9. The chemical reaction for covalent attachment of DOTA to the phage surface is carried out for 16 hours at 35°C. (C) The ^64^Cu^2+^ radiometal is attached through coordinative binding to the DOTA-phage construct for 50 min at 50°C.

Next, we explored the ability of the QFPPKLTNNSML peptide to deliver molecular cargo to sites of ongoing angiogenesis by noninvasive positron emission tomography (PET) imaging. For this purpose we developed a labeling platform employing the phage that displays the QFPPKLTNNSML peptide as a molecularly targeted imaging agent. Using phage as imaging probes and biological nanoparticles in targeting tumors offers an immediate advantage: phage can be covalently attached to numerous labels while simultaneously expressing multiple copies of the tumor-avid peptide [27]. In this study we labeled QFP-phage with ^64^Cu radionuclide via the macrocyclic chelator 1,4,7,10-tetraazacyclododecane-1,4,7,10-tetra acetic acid (DOTA). First, we functionalized the phage particle conducting a coupling chemical reaction between amino groups exposed on the phage surface and the bifunctional p-SCN-Bn-DOTA derivative as illustrated in Figure 5. In a second step we performed a labeling reaction to attach the ^64^Cu^2+^ radiometal to the DOTA-phage-QFP construct. ^64^Cu radiolabeled insertless phage conjugates and ^64^Cu-DOTA complexes were prepared to serve as controls. The chemical modification of the phage surface did not affect cellular binding or phage infectivity (results not shown). Thus phage retained target affinity and biological activity on labeling.

**Figure 6:**
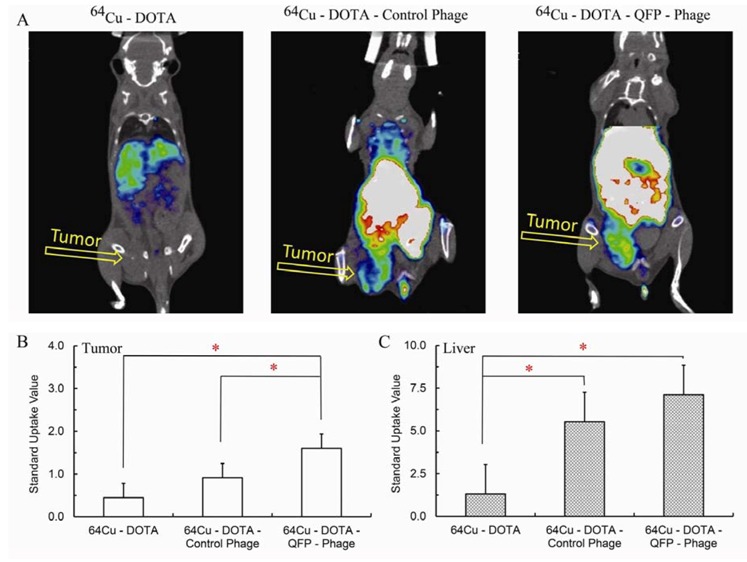
(A) Representative coronal microPET images of mice bearing LLC tumors. Images were acquired 18 hours post-injection of 800 μCi of ^64^Cu-DOTA, ^64^Cu-DOTA-control non-targeted phage, or ^64^Cu-DOTA-QFP-targeted phage. (B) Standard uptake values calculated from the coronal PET images in tumor (mean ± s.e.m., n = 3). (C) Standard uptake values calculated from the coronal PET images in liver (mean ± s.e.m., n=3). P values were determined by Student’s t-test and were considered statistically significant at P < 0.05.

We utilized PET imaging to quantitate noninvasively the ability of the ^64^Cu labeled QFP-phage to bind *in vivo* to its target in angiogenic highly vascularized subcutaneously implanted LLC tumors (n = 3). The labeled phage were injected i.v. into a tumor bearing mouse and the localization of ^64^Cu-DOTA-phage -QFP radiotracer visualized. The distribution of two control radiotracers, ^64^Cu-DOTA and ^64^Cu-DOTA- phage with no peptide insert, was examined in parallel. Representative decay corrected coronal PET images at 18 hours post-injection are shown in Figure 6A. ^64^Cu-DOTA-phage -QFP was able to bind its target *in vivo* thus producing an excellent tumor uptake and contrast in the tumor tissue while the control phage showed little to no accumulation in the tumor. ^64^Cu-DOTA complex revealed a completely different pattern of *in vivo* distribution confirming that the signal from the ^64^Cu-DOTA-phage -QFP is due to the ^64^Cu-DOTA covalently bound in a stable manner to the QFP-phage vector. Figure 6B and C quantify the standard uptake values (SUV) for the tumor and liver, respectively. ^64^Cu-DOTA-phage –QFP has a statistically higher standard tumor uptake value compared to both controls.

As seen in Figure 6 there is a capture of radiolabeled phage by the liver. These observations are consistent with the findings from the phage distribution experiments presented in Figure 4. Both QFP-phage and the insertless control phage are taken up and catabolized by the liver. The fact that liver localization is independent of the presence of a peptide on the surface of the phage clearly indicates that liver uptake is a property of the phage, and not the peptide carried by the phage. Taken together our imaging results reveal that the QFPPKLTNNSML peptide identified by *in vivo* functional selection from a phage display peptide library can deliver molecular cargo to angiogenic tumors. 

Possible limitations may be associated with the synthetic approach employed in this study, *i.e.,* the chemical modification of primary amino groups exposed on the surface of the phage particle. The isothiocyanate of the DOTA reagent can presumably form a thiourea with the side chain primary amine of the lysine residue of the QFP-peptide thus potentially reducing the effective binding affinity of the targeting moiety. This in turn may affect the biodistribution profile of the radioconjugate. Detailed MALDI-TOF MS analysis is currently underway to further characterize the reaction products and identify modification sites. Alternative bioconjugation methods such as covalent attachment of DOTA to the carboxylic reactive groups of the aspartic and glutamic acid residues or to the phenol groups of the tyrosine on the phage surface are warranted in future studies.

The ability to target vasculature for imaging or drug delivery purposes has been an elusive goal. Peptide sequences harnessing endogenous cellular machinery to accomplish this goal represent a new targeting approach that is likely to escape immune surveillance. The studies reported here provide proof-of-principle for this approach. Subsequent refinements using modified peptide sequences coupled to imaging agents and/or biologically active molecules may have clinical utility in a variety of physiologic and pathologic processes where neovascular growth is a critical component.

## 3. Experimental Section

### 3.1. Animals, cell culture, and tumors

Female C57BL/6 mice (Jackson Laboratories, Bar Harbor, ME) 6 to 8 weeks old were used in this study. The animal experiments were approved by the Institutional Animal Care and Use Committee at the University of North Carolina at Chapel Hill.

Human blood outgrowth endothelial cells (HBOEC) were isolated from peripheral blood of consented volunteer donors and cultured as previously described [16,29]. Human umbilical vein endothelial cells (HUVEC) (Lonza, Walkersville, MD) were maintained in EGM-2 Bullet Kit medium (Lonza). Murine Lewis lung carcinoma (LLC) cells (ATCC, Manassas, VA) were grown in Dulbecco’s modified Eagles medium (DMEM) supplemented with 10% fetal bovine serum. Murine bone marrow cells were collected by flushing femoral and tibial bones of black mice with 1 ml cold PBS containing 1 mM EDTA and 0.5% BSA. BM cells were used without further subpopulation enrichment procedures.

To establish tumors, mice were anesthetized by intraperitoneal administration of Avertin (0.02 mL/g). LLC cells were trypsinized, washed three times in DMEM, counted and re-suspended in 500 μL growth factor reduced Matrigel basement membrane matrix (BD Biosciences, Bedford, MA). Approximately 5 × 10^6^ LLC cells were implanted subcutaneously in the inguinal region of each mouse. Controls included 500 μL Matrigel matrix without LLC cells. A control plug was implanted symmetrically to the LLC tumor in animals used for the final round of *in vivo* biopanning.

### 3.2. Peptide library screening

Combinatorial peptide library displayed on the N-terminus of the pIII minor coat protein of bacteriophage M13 was purchased from New England Biolabs (Beverly, MA). The library contains approximately 2.7 × 10^9^ random 12-mer amino acid sequences. Tumor-associated peptide ligands were selected by a combination of *in vitro* and *in vivo* panning protocols. Briefly, for the *in vitro* selection 1 × 10^11^ pfu of the original library were pre-cleared on HUVEC suspension for 2 h at 4 °C. The phage supernatant from the negative selection on HUVEC was incubated for 1 h at 4 °C with HBOEC suspension. Unbound phage pool was washed way, HBOEC-bound phage were eluted, amplified in *E. Coli*, and used for subsequent enrichment cycles as previously described [16]. After three rounds of biopanning on HBOEC, the enriched phage pool, designated as mini-library, was utilized for *in vivo* selection. Two selection schemes were designed and the experiments were conducted in parallel: (i) The mini-library (1 × 10^11^ pfu) was injected i.v. into the tail vein of a LLC tumor bearing mouse or (ii) The mini-library (1 × 10^11^ pfu) was used for labeling freshly isolated BM cells. Phage were allowed to bind to the BM cell suspension in EGM-2 containing 0.5% BSA for 1 h at RT. Unbound phage were washed way and BM-bound phage were administered i.v. in a LLC tumor bearing mouse. In both schemes, 2 hours post-phage injection the mice were perfused through the heart with PBS, the tumors harvested, and processed to elute the tumor-bound phage. Phage from each protocol were amplified separately in *E. coli*, and either were administered i.v. in a LLC tumor bearing mouse, or bound to BM cells and used for the next round of functional selection. The phage panning process was repeated three times for free phage and for BM-bound phage, respectively. All biopanning experiments were carried out 4 days post-tumor implantation. This time point was determined experimentally by monitoring the ability of subcutaneously implanted LLC tumors to recruit BM cells (data not shown).

### 3.3. DNA sequencing

For each *in vivo* screening procedure, fifty individual phage clones from the tumors and the control plugs were randomly picked and the peptide coding inserts were sequenced using -96III sequencing primer, 5’-GCCCTCATAGCGTAACG-3’, (New England Biolabs) following the manufacturer’s instructions. DNA sequencing was performed by the UNC-CH Genome Analysis Facility (Chapel Hill, NC). 

### 3.4. Peptide sequence analysis

For homology identification the primary peptide sequences were analyzed using the Basic Local Alignment Search Tool (BLAST) services provided by NCBI [20]. Non redundant protein database (**nr**) searched with algorithm parameters adjusted for identification of short and nearly exact matches were utilized. Conserved Domain Database (CDD) was searched to find domains conserved across species [24]. The repository for phage derived peptides, MimoDB, was examined to check if the peptide sequences identified here have been found to bind to other targets [17].

### 3.5. Characterization of QFP-phage clone binding to LLC tumors

The selectivity of QFP-phage towards LLC tumors and phage distribution in blood, liver and heart were analyzed using the same protocols and conditions as for the mini-library screening.

### 3.6. Phage modification

QFP-phage and control insertless phage were amplified to a high titer for labeling with ^64^Cu radionuclide via the macrocyclic bifunctional chelator 2-(4-isothiocyanatobenzyl)-1,4,7,10 tetraa-zacyclododecane-1,4,7,10-tetraacetic acid (p-SCN-Bn-DOTA) (Macrocyclics, Dallas, TX). The isothiocyanate functionality of the p-SCN-Bn-DOTA reacts with primary amino groups on the phage coat protein pVIII to produce a covalent attachment of DOTA to the phage surface [30]. To carry out this functionalization procedure, phage (1 × 10^11^ pfu) were re-suspended in 400 μL conjugation buffer (carbonate-bicarbonate buffer, pH = 9) and 4 μL p-SCN-Bn-DOTA (100 mM stock) were added. The conjugation reaction was conducted overnight at 35 °C. DOTA-phage intermediate was separated from the unreacted DOTA through a 50 K microcone filter by centrifugation at 14,000g for 10 minutes. DOTA-phage conjugates were re-suspended in 400 μL 0.1M sodium acetate buffer (pH = 5.5) and incubated with ^64^CuCl_2_ (2 mCi per reaction, decay corrected) for 50 minutes at 50^o^C. Unbound ^64^Cu^2+^ was removed by ultracentrifugation as described above. ^64^Cu labeled phage were reconstituted in sterile PBS. Activity was determined using a γ-counter (Packard) immediately before injecting into mice. Complexes of DOTA-phage with cold CuCl_2_ were prepared to test phage infectivity and target binding upon labeling using protocols described above for chemically unmodified phage particles. 

### 3.7. MicroPET imaging

MicroPET scans and image analysis were performed on a GE Explore Vista microPET/CT rodent scanner. At seven days post-implantation, mice bearing subcutaneous LLC tumors (tumor volume approx. 1 cm^3^) were injected with 800 μCi of radiotracer ^64^Cu – DOTA (n = 3), ^64^Cu –DOTA-control phage (n = 2) or ^64^Cu –DOTA-QFP-phage (n = 3) into the tail vein at a volume 150 μL. Ten minute microPET static scans were acquired 18 hours post-contrast injection under isoflurane anesthesia. Images were reconstructed and the radioactivity concentration within tumor and liver was converted into standard uptake values. Regions of interest (ROI) for the tumor were selected from the right lateral (actively perfused shell of the tumor), avoiding the (necrotic/lower intensity) regions. Location of the ROI within tumor margins was confirmed by comparison with the registered CT image. The liver ROI was selected in the uniform central region of the right lobe, placed away from the edges to avoid partial volume effect from the large voxel size in PET images. 

## 4. Conclusions

Using a combination of *in vitro* and *in vivo* functional screening protocols we have isolated the QFPPKLTNNSML peptide that is selective for circulating bone marrow derived cells homing to angiogenic LLC tumors. We have demonstrated the ability of the QFPPKLTNNSML peptide to deliver molecular cargo to sites of ongoing angiogenesis by noninvasive PET imaging. The selection strategy we have described in this report can be easily extended to target other malignancies that recruit bone marrow derived cells to sustain growth of new blood vessels.
